# Importance of extracorporeal membrane oxygenation (ECMO) in congenital heart diseases: a systematic review

**DOI:** 10.1186/s43044-025-00667-7

**Published:** 2025-07-11

**Authors:** Muhammad Shaheer Bin Faheem, Ahmed Ali Khan, Shamikha Cheema, Muzamil Akhtar, Danish Ali Ashraf

**Affiliations:** 1 Karachi Institute of Medical Sciences, KIMS, Karachi, Pakistan; 2 Foundation University Medical College, Islamabad, Pakistan; 3 King Edward Medical University, Lahore, Pakistan; 4 Gujranwala Medical College, Gujranwala, Pakistan

## Abstract

**Background:**

Congenital heart diseases (CHDs) represent a significant healthcare challenge with incidence rates of 17.9 per 1000 live births. Extracorporeal membrane oxygenation (ECMO) has become an invaluable therapeutic option providing essential aid to support both cardiac as well as pulmonary failure.

**Methods:**

A systematic search was performed using PubMed, Embase, and Scopus from 2000 till date. Observational studies involving pediatric patients with CHD undergoing cardiac surgery using ECMO were included. The main outcomes were to determine short-term mortality, weaning off ECMO, complications, hospital and ICU length of stay, and indications for ECMO. Assessment of the risk of bias of included studies was done by Newcastle–Ottawa scale.

**Results:**

24 retrospective observational studies, encompassing 1,658 patients, were ultimately included in our review. The overall incidence of mortality across these studies was 45.2%. Successful weaning from ECMO was achieved in 73.9% of patients. The most frequently reported complications included bleeding, which affected 42.9% of patients, renal failure in 42.5%, and sepsis in 27.5%. The mean duration of hospital stay was 47.8 ± 41.1 days, while the mean length of stay in the ICU was 33.4 ± 32.6 days.

**Conclusions:**

ECMO benefits pediatric heart patients but comes with risks like bleeding and high mortality. Percutaneous techniques can reduce complications; more research on minimally invasive approaches is needed.

## Introduction

Congenital heart disorders (CHDs) pose a notable healthcare burden having a global incidence rate of 17.9 per 1000 live births with the highest incidence rate in evolving countries and lowest in developed countries. CHD is any anatomical cardiac anomaly and its major vessels present at birth [1]. Although significant progress has been made in cardiovascular surgery and medicine in recent decades permitting most diseased individuals to attain adulthood, CHDs remain the leading cause of mortality and morbidity worldwide [2].

Despite the advancements in surgery and pre- and postoperative rehabilitation, some patients continue to experience severe cardiovascular and pulmonary distress, particularly in the early stages of infancy or following corrective procedures. For these patients, extracorporeal membrane oxygenation (ECMO) is best therapeutic option. More than 36,000 children have been put on ECMO so far and as the innovation progresses so does the demand for this life-saving aid [3]. ECMO offers artificial cardiovascular aid in the event of either single or combined circulatory failure. Given its evolving and expanding scope, physicians need to be familiar with the scope and advancements of this novel technology [4].

ECMO can be employed in two main setups: venovenous (VV) ECMO for right ventricular failure from underlying respiratory failure and venoarterial (VA) ECMO for combined dysfunction of both the right and left ventricles. Both indirectly support the RV by reducing preload and RV wall stress thereby improving oxygen saturation to coronary vasculature [5]. ECMO is a device that can replace the lungs and heart. To use ECMO, physicians insert special tubes called cannulas into any of the patients’ major veins in the torso, thighs, or neck (cannulation). The ECMO machine then pulls blood from the patient’s body. The blood then passes through an artificial respirator, which adds oxygen and removes carbon dioxide, just like healthy lungs do. Oxygenated blood is then pumped back into the patient’s body with the same force as a healthy heart. A perfusionist (a specially trained healthcare professional) controls the settings of the ECMO machine to provide the patient with appropriate levels of cardiac and pulmonary [6]. Thus, in children with CHD ECMO is utilized to improve both the pulmonary and cardiovascular outcomes not only to treat the underlying illness but also to provide time for further investigations and to temporarily relieve their symptoms while they wait for other medical treatments. Patients having teratology of Fallot, extreme postoperative hypoxia, low cardiac output syndrome, cardiac failure, transposition of great vessels, and total anomalous pulmonary venous connection can utilize ECMO [7]. VV ECMO depends on the patient’s intrinsic cardiac output for circulation and provides respiratory support by extracting deoxygenated blood and reinfusing oxygenated blood into the venous system. On the other hand, VA-ECMO returns oxygenated blood to arterial system (5].

To sum up, given the critical role ECMO plays in the management of congenital heart diseases, a thorough understanding of its effectiveness, safety, consequences, challenges, and outcomes is essential. This systematic review aims to examine the current literature on the use of ECMO in congenital heart diseases. By combining the results from other studies, this systematic review seeks to analyze the existing literature on the use of ECMO in hereditary cardiac disorders and evaluate postoperative ECMO assistance including survival rate, weaning off from ECMO, and associated complications. This contributes to a thorough understanding of the functions of ECMO in congenital heart disorders, which will guide further research to advance pediatric surgery.

## Methodology

### PICO criteria

In pediatric patients with congenital heart diseases (CHD) undergoing cardiac surgery (P), does the use of venoarterial ECMO (I) reduce mortality and complications (O)?

### Literature search strategy

According to PRISMA statement, authoritative data bases like PubMed, Embase, and Scopus were used. Heart defects, congenital heart defects, ECMO, extracorporeal membrane oxygenation, extracorporeal life support, ECLS, and mechanical circulatory support were the search terms used. A systematic search was conducted using a combination of keywords, Boolean operators, and Medical Subject Heading (MeSH) terms, from inception till up to date to identify relevant studies comprehensively. The complete search strategy for PubMed is also available in the supplementary material. 

### Eligibility criteria

#### Inclusion criteria

This study included pediatric patients less than 18 years old with congenital heart diseases requiring ECMO support after cardiac surgery. Only observational studies with sample size of greater than 10 were included.

#### Exclusion criteria

Studies using extracorporeal life support organization (ECMO) registry were excluded due to overlapping population. Moreover, articles with sample size of ≤ 10 and publication before 2000 were also excluded. The main reason was that: (1) The research methodologies were not so rigorous before 2000 as they are now and (2) we wanted to incorporate latest and accurate information in our review.

### Search results

**Fig. 1:**
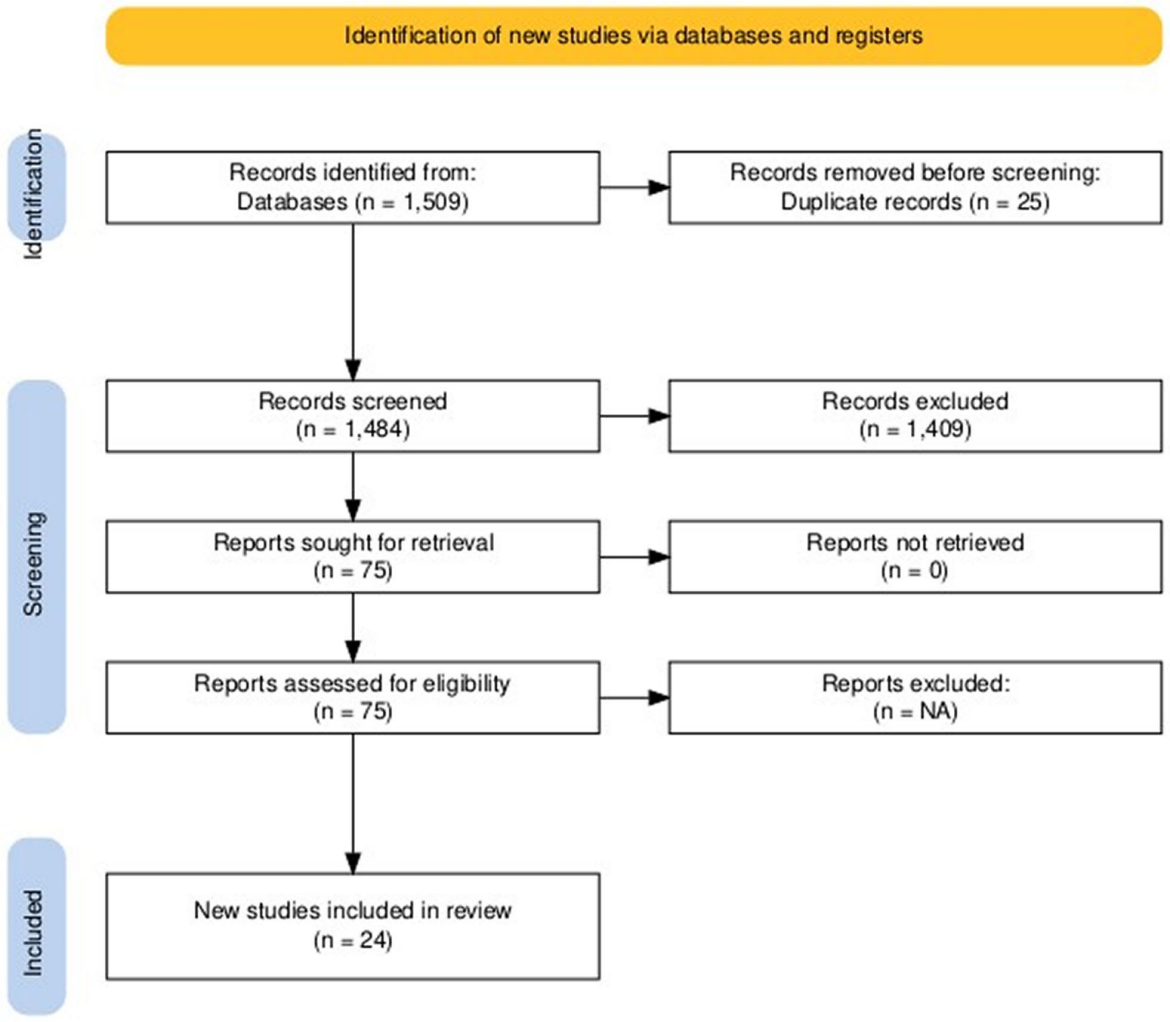
PRISMA flow diagram

In accordance with the inclusion and exclusion criteria, two reviewers (M.F. and M.A.) screened the titles and abstracts of all studies for preliminary screening using the internet software Rayyan. Any discrepancy was resolved by the third reviewer (D.A.). After removing 25 duplicates, a total of 1509 studies were found. Out of these, 1484 underwent title and abstract screening, and 1409 of them were excluded. Then full texts of 75 studies were read precisely and 51 studies were excluded on basis of following reasons: (1) studies using ECMO registry and (2) sample size less than or equal to 10. Finally, our systematic review included 24 studies. The screening process is illustrated in Fig. 1 [8].

### Data extraction

Data extraction involved reviewing the full texts of all 24 studies by MF, with any discrepancies resolved by DA. One reviewer extracted the study characteristics including study design, origin, sample size, gender distribution, mean age, mean weight, total ECMO duration, total hospital LOS, total ICU LOS, and complications. To address heterogenicity, we omitted meta-analyses and rechecked all the extracted data twice.

### Outcomes

The main outcomes were to assess short-term mortality (defined as 30-day mortality or in-hospital mortality), weaning off ECMO (defined as successful discontinuation of ECMO support without the need for reinitiation within 24– 48 h), complications, hospital and ICU length of stay, and common indications for ECMO. To avoid high heterogenicity due to different study designs and lack of common outcomes in studies, meta-analyses were not conducted.

### Assessment of risk of bias

One reviewer (A.A.) independently assessed the risk of bias of included studies using the Newcastle–Ottawa scale. This tool uses domains like sampling plan, statistical analysis description, and outcomes.

## Results

### Study selection and characteristics

A comprehensive literature search using the search strategy yielded 1,509 articles. After eliminating duplicates, 1,484 studies underwent initial screening. Following a thorough review of titles and abstracts, 75 articles were selected for full-text evaluation. Ultimately, 24 articles were included in the final review. The study selection process is detailed in Fig. 1. All selected studies were retrospective observational studies. These studies were conducted across nine countries: the USA (*n* = 9), China (*n* = 6), Japan (*n* = 2), Saudi Arabia (*n* = 2), Mexico (*n* = 1), India (*n* = 1), Turkey (*n* = 1), and Australia (*n* = 1).

The cumulative cohort comprised 1,658 patients with congenital heart disease (CHD) who received post-cardiotomy VA-ECMO support. The basic characteristics of the included literature are detailed in Table 1.

**Table 1:** Risk of bias assessment using the NOS

NOS domain	A. Selection (maximum of four stars)				B. Comparability (maximum of two stars)	C. Outcome (maximum of three stars)			Total (maximum of nine stars)
Study (year)	1. Representativeness of the exposed cohort	2. Selection of the non-exposed cohort	3. Ascertainment of exposure	4. Demonstration that outcome of interest was not present at start of study (no bone disease at start of study)	1. Comparability of cohort on the basis of the design or analysis	1. Assessment of outcome	2. Was follow-up long enough for outcomes to occur	3. Adequacy of follow-up of cohorts	
Basgoze et al. (2022)	☆^a^	★	★	★ (mentions excluding patients with outcome prior to surgery)	☆☆^d^	★	★	★	6
Yu et al. (2021)	☆^a^	★	★	★ (mentions excluding patients with outcome prior to surgery)	☆☆^d^	★	★	★	6
Taka et al. (2021)	☆^a^	★	★	☆^c^	☆☆^d^	★	★	★	5
Jin et al. (2020)	☆^a^	★	★	☆^c^	☆☆^d^	★	★	★	5
Dohain et al. (2019)	☆^a^	★	★	☆^c^	☆☆^d^	★	★	★	5
Jesus-Brugman et al. (2019)	☆^a^	★	☆^b^	☆^c^	☆☆^d^	☆^e^	★	★	3
Yang et al. (2019)	☆^a^	★	★	☆^c^	☆☆^d^	★	★	★	5
Chrysostomou et al. (2013)	☆^a^	★	★	☆^c^	☆☆^d^	★	★	★	5
Thourani et al. (2006)	☆^a^	★	☆^b^	☆^c^	☆☆^d^	☆^e^	★	★	3
Shah et al. (2005)	☆^a^	★	☆^b^	☆^c^	☆☆^d^	☆^e^	★	★	4
ElMahrouk et al. (2019)	☆^a^	★	★	☆^c^	☆☆^d^	★	★	★	5
Guo et al. (2018)	☆^a^	★	★	☆^c^	☆☆^d^	★	★	★	5
Alsoufi et al. (2014)	☆^a^	★	★	★ (mentions excluding patients who had been under ECMO support prior to study)	☆☆^d^	★	★	★	6
Chauhan et al. (2011)	☆^a^	★	★	★ (mentions excluding patients who had been under ECMO support prior to study)	☆☆^d^	★	★	★	6
Kumar et al. (2010)	☆^a^	★	★	☆^c^	☆☆^d^	★	★	★	5
Baslaim et al. (2006)	☆^a^	★	★	☆^c^	☆☆^d^	★	★	★	5
Derby et al. (2007)	☆^a^	★	★	☆^c^	☆☆^d^	★	★	★	5
Zhao et al. (2008)	☆^a^	★	★	☆^c^	☆☆^d^	★	★	★	5
Lv et al. (2016)	☆^a^	★	★	★ (mentions excluding patients with outcome prior to surgery)	☆☆^d^	★	★	★	6
Sznycer-Taub et al. (2016)	☆^a^	★	★	★ (mentions excluding patients who had been under ECMO support prior to study)	☆☆^d^	★	★	★	6
Bezerra et al. (2022)	☆^a^	★	★	☆^c^	☆☆^d^	★	★	★	5
Vargas-Camacho et al. (2020)	☆^a^	★	★	★ (mentions excluding patients who were predisposed to outcome due to comorbidities and other confounding factors)	☆☆^d^	★	★	★	6
Asano et al. (2019)	☆^a^	★	★	★ (mentions excluding patients who had been under ECMO support prior to study)	☆☆^d^	★	★	★	6
Crawford et al. (2024)	★ (it is a registry-based study that encompasses data from hospitals of 2 countries)	★	★	☆^c^	☆☆^d^	★	★	★	6

If a star was not awarded, the reason is indicated by a superscript letter (e.g., ☆^e^) next to the empty star in the table. The corresponding explanation can be found in the footnotes. ^a^It is single-centered study; potential for selection bia. ^b^The investigators do not mention collecting data using secure records/structured interviews. ^c^The article does not mention exclusion criteria; or excluding patients on ECMO support prior to study. ^d^The study only mentioned that differences in demographics between the cohorts were non-significant (*p* > 0.05); no propensity score matching done. ^e^The study does not mention collecting data using secure records/structured interviews. Therefore, the risk of reporting bias may be present

### Risk of bias assessment

The methodological quality and risk of bias in the studies included in this review were assessed using the Newcastle–Ottawa scale (NOS). A significant variation in methodological rigor was observed, with studies scoring between 3/9 and 6/9 stars, indicating a moderate to high risk of bias within the study pool. Of the 24 studies included in the review, 23 were single-centered retrospective cohort studies and 16 out of 24 studies did not mention excluding patients on prior ECMO support or those with outcomes before the surgical procedure. This points to a high risk of selection bias in the results and raises concerns about reporting accuracy. A lack of clearly defined exclusion criteria, particularly for patients already on ECMO, may have led to population heterogeneity.

Most studies in the review did not perform matching or propensity score analysis to account for demographic differences between cohorts and adjust for confounders. Furthermore, contributing to selection bias, these methodological limitations may impact the reliability and generalizability of the findings of this review. As such, findings should be interpreted cautiously, and there remains a need for well-designed, multicenter studies with standardized reporting practices.

Additionally, 3 out of 24 studies failed to mention the source of their data (e.g., registries, medical records, patient registers, charts, etc.). These studies were not awarded a star in two NOS domains—ascertainment of exposure and assessment of outcome—due to the potential risk of reporting bias. This is presented in Tables 1 and 2

**Table 2:** Summary of study characteristics and outcomes in the included studies

	Study characteristics						ECMO characteristics and outcomes in CHD							ECMO complications			
Sr no	Author name (year)	Origin	Total population (n)	Male/Female ratio (n)	Neonates/Infants ratio (n)	Mean age ± SD (days)	Indications for ECMO	Types of CHD for which ECMO was performed	Mean duration of ECMO ± SD (days)	Mean hospital LOS ± SD (days)	Mean ICU LOS ± SD (days)	Mortality rate (%)	Weaning off from ECMO (%)	Hemorrhagic (n)	Infectious (n)	Organ dysfunction—Renal failure (n)	Organ dysfunction—Neurological events (n)
1	Basgoze et al. (2022)	Turkey	109	63/46	50/37	307.4 ± 627.1	Low cardiac output; Respiratory failure	Norwood procedure = 18; Left ventricular outflow tract and/or aortic reconstruction = 16; Arterial switch operation = 12; Total correction of Tetralogy of Fallot = 10	Survivors: 4.5 ± 2.3	60.5 ± 63.5	54.4 ± 52.9	75.2	49.5	24	34	87	32
2	Yu et al. (2021)	China	23	15/8	23/0	9.1 ± 8.0	Low cardiac output syndrome; Extracorporeal cardiopulmonary resuscitation (ECPR)	Ventricular septal defect and Coarctation of Aorta = 10; Transposition of the great arteries = 8	Survivors: 6.0 ± 3.8	24.3 ± 9.8	18.0 ± 7.5	NR	78.3	16	NR	5	8
3	Taka et al. (2021)	Japan	37	18/19	10/0	303.1 ± 176.0	NR	Single ventricle physiology = 18; Hypoplastic left heart syndrome = 10	6.7 ± 3.0	NR	NR	40.5	78	NR	NR	NR	7
4	Jin et al. (2021)	China	85	52/33	3/38	386.6 ± 186.7	Failure to wean from cardiopulmonary bypass = 42; Extracorporeal cardiopulmonary resuscitation (ECPR); Low cardiac output syndrome	Transposition of the great arteries	6.0 ± 3.4	49.0 ± 30.6	32.4 ± 25.2	52.9	70.5	59	NR	53	11
5	Dohain et al. (2019)	Saudi Arabia	30	NR	NR	277.7 ± 144.1	Failure to wean from cardiopulmonary bypass = 14; Low cardiac output syndrome = 6; Extracorporeal cardiopulmonary resuscitation = 10	12 single ventricle repairs; 18 biventricular repairs	5.0 ± 0.9	46.1 ± 40.5	36.8 ± 35.2	60.0	66.7	4	NR	14	10
6	De Jesus-Brugman et al. (2020)	USA	25	NR	25/0	NR	Failure to wean from cardiopulmonary bypass; Low cardiac output syndrome; Extracorporeal cardiopulmonary resuscitation; Hypoxemia	Norwood procedure	4.5 ± 5.0	44.9 ± 25.1	24.0 ± 17.3	72.0	NR	NR	NR	NR	7/25
7	Yang et al. (2019)	China	56	NR	NR	1461.1 ± 776.2	NR	Transposition of the great arteries; Tetralogy of Fallot	3.6 ± 3.5	21.6 ± 14.5	12.4 ± 8.4	39.3	67.9	30	NR	10	9
8	Chrysostomou et al. (2013)	USA	95	57/38	NR	NR	NR	Single ventricle physiology; Biventricular physiology	NR	NR	NR	27.4	NR	17	NR	21	11
9	Thourani et al. (2006)	USA	27	19/8	NR	139.3 ± 183.6	Failure to wean from cardiopulmonary bypass; Extracorporeal cardiopulmonary resuscitation; Low cardiac output syndrome	Single ventricle physiology; Biventricular physiology	6.3 ± 8.4	41.0 ± 45.5	NR	NR	NR	NR	NR	NR	NR
10	Shah et al. (2005)	USA	84	NR	NR	128.0 ± 304.0	Failure to wean from cardiopulmonary bypass; Extracorporeal cardiopulmonary resuscitation; Low cardiac output syndrome	Single ventricle physiology; Biventricular physiology; Hypoplastic left heart syndrome	NR	NR	NR	63.1	NR	36	NR	41	4
11	ElMahrouk et al	NA	113	67/46	NR	90.0 ± 303.5	Failure to wean from cardiopulmonary bypass; Extracorporeal cardiopulmonary resuscitation; Low cardiac output syndrome; Pulmonary failure	Single ventricle physiology; Biventricular physiology	4.0 ± 3.7	NR	NR	62.8	46.9	78	35	41	18
12	Guo et al. (2018)	China	11	8/3	4/0	168.2 ± 143.8	NR	NR	3.0 ± 4.0	NR	NR	NR	NR	6	2	6	4
13	Alsoufi et al. (2014)	USA	100	63/37	33/46	73.0 ± 186.7	Failure to wean from cardiopulmonary bypass; Extracorporeal cardiopulmonary resuscitation; Low cardiac output syndrome	Single ventricle physiology; Biventricular physiology	4.0 ± 1.5	35.9 ± 22.6	NR	63.0	NR	75	21	55	17
14	Chauhan et al. (2011)	India	94	NR	NR	53.0 ± 238.5	NR	Transposition of the great arteries	1.8 ± 3.8	NR	NR	35.1	NR	NR	23	14	NR
15	Kumar et al. (2010)	USA	58	31/27	NR	74.0 ± 50.0	Failure to wean from cardiopulmonary bypass; Extracorporeal cardiopulmonary resuscitation; Low cardiac output syndrome; Hypoxia; Cardiac arrest	Single ventricle physiology; Biventricular physiology; Hypoplastic left heart syndrome	6.0 ± 2.5	NR	NR	58.6	67.2	NR	9	18	17
16	Baslaim et al. (2006)	Saudi Arabia	26	NR	NR	499.2 ± 1092.0	NR	Single ventricle physiology; Biventricular physiology	NR	NR	NR	53.9	NR	17	4	8	5
17	Derby et al. (2007)	USA	37	NR	NR	161.8 ± 118.5	NR	Single ventricle physiology; Biventricular physiology; Transposition of the great arteries	2.8 ± 3.8	NR	NR	54.1	19.0	9	3	4	8
18	Zhao et al. (2008)	China	20	NR	NR	NR	NR	NR	5.0 ± 4.0	NR	NR	NR	75	7	NR	6	NR
19	Lv et al. (2016)	China	42	24/18	NR	507.3 ± 172.0	NR	Tetralogy of Fallot; Pulmonary atresia	5.8 ± 2.5	NR	NR	47.6	NR	NR	NR	17	NR
20	Sznycer-Taub et al. (2016)	USA	93	54/39	70/23	7.0 ± 2.5	Failure to wean from cardiopulmonary bypass; Extracorporeal cardiopulmonary resuscitation; Low cardiac output syndrome; Hypoxia; Cardiac arrest	NR	5.3 ± 2.0	NR	27.6 ± 21.1	37.6	NR	NR	NR	35	39
21	Bezerra et al. (2022)	USA	33	21/12	NR	25.7 ± 17.0	NR	NR	5.0 ± 7.5	NR	NR	66.7	NR	NR	NR	NR	22
22	Vargas-Camacho et al. (2020)	Mexico	11	7/4	7/0	NR	NR	Transposition of the great arteries; Pulmonary stenosis; Tetralogy of Fallot	6.3 ± 4.9	NR	NR	NR	NR	NR	4	4	1
23	Asano et al. (2019)	Japan	73	43/26	NR	453.6 ± 779.3	NR	NR	8.3 ± 4.4	73.8 ± 48.0	NR	39.2	84.8	24	39	55	3
24	Crawford et al. (2024)	Australia & New Zealand	376	NR	NR	22.0 ± 19.9	NR	Single ventricle physiology; Biventricular physiology	NR	NR	NR	22.6	85.6	129	NR	NR	22

### Outcomes

#### Short-term mortality

Short-term mortality was defined as 30-day mortality or in-hospital mortality. All studies reported data on short-term mortality. Of the 1,658 patients with CHD receiving post-cardiotomy VA-ECMO support, 750 deaths were recorded, yielding an overall mortality rate of 45.2%.

#### Weaning off ECMO

Weaning off ECMO was defined as the successful discontinuation of ECMO support without the need for reinitiation within 24 to 48 h, depending on the criteria used in the individual studies. Thirteen studies reported data on weaning outcomes, involving a total of 991 patients. Of these, 732 patients (73.9%) were successfully weaned off ECMO.

#### Complications

Complications associated with VA-ECMO support were reported in 23 studies. Hemorrhagic complications like bleeding affected 42.9% of patients. Organ dysfunction included complications like renal failure (42.5%) and neurological events (17.3%). Infectious complications included sepsis which occurred in 27.5% patients.

#### Hospital and ICU length of stay

The mean hospital length of stay, reported in nine studies involving 534 patients, was 47.7 ± 41.1 days. The mean ICU length of stay, reported in seven studies involving 421 patients, was 33.4 ± 32.8 days.

#### Common indications for ECMO

The studies identified several common indications for VA-ECMO support, including low cardiac output, failure to wean from cardiopulmonary bypass, cardiac arrest, and respiratory failure.

## Discussion

ECMO machines serve as an invaluable life support system for both pediatric and adult patients suffering from refractory cardiopulmonary dysfunction [9]. These machines use a pump and oxygenator to replace the function of the heart and lungs when conventional measures or CPR fail to achieve a return of spontaneous circulation (ROSC) [10]. In our systematic review of literature, we aim to synthesize findings from the latest studies examining the safety and efficacy of this modality in pediatric patients with congenital heart diseases. VA-ECMO was frequently indicated in patients with reduced cardiac output, cardiac arrest, and failure to wean from the heart–lung bypass in studies included in this review. Data from of a 10-year-long study conducted by Leite et al. also revealed that VA-ECMO can be a life-saving intervention in post-acute coronary syndrome patients developing cardiogenic shock, myocarditis, and post-valvular surgery [11]. Understanding the common triggers for ECMO initiation may guide future protocols leading to timely intervention and better prognostic outcomes.

There are many factors which influence the weaning success rates from VA-ECMO. These include old age, CKD, hypertension, and support extending for a period over approximately 7 days [12]. Our review showed that weaning success rate among patients receiving VA-ECMO was promising (73.9%) which suggests that irrespective of the high-risk status of the patients in the cohort, most of them can be successfully liberated from ECMO. Higher weaning success rates (100%) have also been seen in procedures involving modified post-closure techniques [13]. While these liberation rates are promising, these predictors should be considered to optimize patient selection and ECMO management strategies.

Patients included in this review stayed in the hospital for approximately more than a month (33.4–47.7 days on average). Extended hospital stays of patients on ECMO support can be resource-intensive for healthcare systems. Data from a retrospective analysis of over seventeen thousand hospitalizations in the US over the years 2019 and 2020 were recently published. ECMO procedures not only had a significantly higher mortality rate as compared to non-ECMO procedures, but also a higher cost per hospitalization. ECMO involves over 10 procedures which added up to over USD 967 000 per hospitalization as compared to only USD 52000 for non-ECMO hospitalizations [14]. One of the main factors increasing the financial burden and hospital charges for patients includes vascular complications during ECMO therapy [15].

VA-ECMO serves as a life-saving procedure; however, data from the included studies showed that patients undergoing receiving ECMO were at a greater risk of complications owing to patient transport, general anesthesia, and intubation procedures required prior to closure of access site. Kazui et al. in their study report a strong positive correlation of increased duration of ECMO with bleeding events, highlighting the need for more vigilant monitoring of these patients [16]. Organ failure involving the kidneys and brain was frequently observed in the included studies and significantly increases morbidity in ECMO patients [17]. Additionally, infection rates are higher in patients experiencing prolonged hospital stays due to these complications. The presence of infections significantly correlates with in-hospital mortality, highlighting the importance of infection control measures [18].

The procedure was also associated with a high short-term mortality rate in post-cardiotomy CHD patients which can be attributed to several factors. Patients with CHD often present with complex anatomical challenges such as incomplete repair of anatomical malformations after surgery [19] and physiological challenges, such as single ventricular physiology, which significantly increase mortality risk [20]. Additionally, CHD patients have higher pre-ECMO lactate levels which are a strong marker of inadequate tissue perfusion and anaerobic metabolism. This leads to ischemic damage of the tissues and increases morbidity and mortality [19, 21]. ECMO is often initiated in emergency situations like cardiac arrest, and postoperative complications which inherently carry increased risk of mortality [22].

Complication rate associated with ECMO can be drastically minimized by employing strategies to optimize selection of patients who will be receiving support through ECMO. First, identification of key clinical indicators can guide timely initiation of ECMO which include failure to wean from cardiopulmonary bypass, low cardiac output, and hypoxia [23]. Declining renal function, high pre-ECMO serum lactate and bilirubin levels are associated with higher mortality in ECMO patients and hence can help in risk-based stratification of patients who are candidates for ECMO [24]. As previously highlighted, inadequate repair of anatomical malformations increases ECMO dependency in CHD patients. These structural defects can be identified earlier by employing advanced imaging modalities (e.g., cardiac catheterization) which can significantly reduce reliance of CHD patients on ECMO for life support [25]. Finally, decision-making regarding ECMO initiation and management can be optimized by encouraging collaborations by physicians from multiple specialties, ensuring that all clinical aspects are considered [25].

Betz et al. (2023), in their case series, highlight the effectiveness of using a percutaneous decannulation technique. This mitigates the risk of complications in VA-ECMO patients and ensures safe, bedside closure in the ICU—while the patient is awake [26]. These findings are further supported by a Peng et al. (2024) study on the efficacy and safety of a percutaneous, bedside, 3 stitch suture technique [27]. Complications during VA-ECMO closure may be minimized by incorporating percutaneous procedures as compared to surgical techniques [28]. Investigations focusing on comparing minimally invasive approaches with surgical methods and strategies to minimize these adverse events are warranted. Such complications prolong hospitalization periods, thus burdening the healthcare system and patients as well.

### Limitations

Variability in surgical approaches, ECMO protocols, and institutional expertise across included studies introduces heterogeneity. These differences may have influenced patient outcomes and should be standardized in future prospective investigations.

## Conclusions

Further research should prioritize multicentric RCTs comparing outcomes of ECMO supplemented with minimally invasive percutaneous cannulation and closure techniques versus conventional open surgical approaches. These studies should assess short- and long-term mortality, vascular complication rates, neurological outcomes, and cost-effectiveness. Additionally, standardization of ECMO protocols across all hospitals will help reduce the inconsistencies in clinical outcomes across different populations.

## Data Availability

Data is provided within the manuscript.

## Abbreviations

CHDs: Congenital heart diseases
ECMO: Extracorporeal membrane oxygenation
VV: Venovenous
VA: Venoarterial
MeSH: Medical subject heading
NOS: Newcastle–Ottawa scale
ROSC: Return of spontaneous circulation
